# Regression analysis of topological indices for predicting efficacy of Alzheimer’s drugs

**DOI:** 10.1371/journal.pone.0309477

**Published:** 2024-11-01

**Authors:** Tahreem Ashraf, Nazeran Idrees, Melaku Berhe Belay

**Affiliations:** 1 Department of Mathematics, Government College University, Faisalabad, Pakistan; 2 Nanotechnology Center of Excellence, Addis Ababa Science and Technology University, Addis Adaba, Ethopia; 3 Mathematics, Physics and Statistics Division, Addis Ababa Science and Technology University, Addis Adaba, Ethopia; GLA University, INDIA

## Abstract

Alzheimer’s Disease(AD) is the most common type of dementia. It is a progressive disease beginning with mild memory loss and possibly leading to loss of the ability to carry on a conversation and respond to the environment. This study investigates the relationship between the chemical structure of potential AD drugs and their therapeutic efficacy using Multi-Criteria Decision-Making (MCDM) techniques including The approach for Order Preference by Similarity to Ideal Solution (TOPSIS) and Simple Additive Weighting (SAW) method. A comprehensive dataset comprising molecular descriptors and corresponding pharmacological properties, i.e., melting point, boiling point, molecular weight and density of AD drugs was compiled from diverse sources. Topological indices were calculated to capture the structural characteristics of these compounds. Application of TOPSIS and SAW through Entropy method helps obtain optimal drugs for curing AD. Quantitative Structure Property Relationships (QSPR) analysis has been done between properties and topological indices of AD’s drug structures. Results revealed significant relations between specific topological indices and drug efficacy, providing insights into the structural features crucial for AD treatment efficacy. This approach offers a promising avenue for rational drug design and optimization in the quest for novel AD therapeutics.

## 1. Introduction

Alzheimer’s Disease (AD) stands as a pervasive neurodegenerative disorder characterized by progressive cognitive decline, memory loss, and functional impairment, affecting millions worldwide [1]. Despite extensive research efforts, effective pharmacological interventions for AD remain elusive, with existing treatments providing only symptomatic relief and limited efficacy in halting disease progression [2]. The complexity of AD pathophysiology, including mechanisms such as amyloid-beta (*αβ*) aggregation, tau protein hyperphosphorylation, and cholinergic neurotransmitter dysfunction, underscores the challenge of developing successful therapeutic strategies [3].

In recent years, advancements in computational chemistry and bioinformatics have facilitated the exploration of Quantitative Structure Property Relationships (QSPRs) in drug discovery and development. Topological indices, derived from molecular graph theory, offer a means to quantify the structural features of chemical compounds and predict their pharmacological properties [4]. Utilizing topological indices in regression analysis provides a systematic approach to elucidate the relationship between molecular structure and drug efficacy, offering insights into the design and optimization of novel therapeutics [5].

This study aims to investigate the potential of topological indices in predicting the efficacy of AD drugs through regression analysis. By leveraging a comprehensive dataset encompassing molecular descriptors and pharmacological activities of AD drugs, we seek to identify key structural determinants associated with therapeutic efficacy. Understanding the structural requirements for effective AD treatment could inform rational drug design strategies and accelerate the development of next-generation therapeutics targeting this devastating disease.

QSPRs describe the relationship between the chemical structure of a compound and its pharmacological activity [3]. Understanding QSPRs is crucial in drug discovery and development for optimizing therapeutic efficacy and minimizing adverse effects [4].

A graph is a mathematical structure composed of vertices (nodes) and edges (links) that connect pairs of vertices. Graphs are widely used to represent relationships between objects or entities in various domains. The degree of a vertex in a graph is the number of edges incident to that vertex [6]. Topological indices are numerical descriptors derived from molecular graph theory that quantify the structural features of chemical compounds. They provide insights into the molecular structure and properties of compounds, facilitating drug design and optimization [5].

This study employed Multi Criteria Decision Making (MCDM) techniques such as The approach for Order Preference by Similarity to Ideal Solution (TOPSIS) and Simple Additive Weighting (SAW) to analyze the properties of various drug structures of AD. It represents the first instance of using these specific MCDM methods to rank any disease structures. TOPSIS, renowned for its ability to assess decision-making problems quantitatively and qualitatively, offers superior accuracy and efficiency in addressing real-world issues compared to other MCDM techniques. Moreover, it stands out for its simplicity, logical approach, active working speed, and capability to express comparative performance for each option in a straightforward numerical manner. As weighing criteria, Entropy method is employed, which maintains the original data’s relative order through a proportional linear transformation. This method is particularly useful when dealing with complex decision-making problems where multiple criteria need to be considered simultaneously. It helps decision-makers prioritize criteria based on their relative importance, leading to more informed and objective decisions. Meanwhile, the SAW method necessitates normalizing the decision matrix to a scale compatible with all other existing rankings.

However, there are certain limitations to TOPSIS and SAW. In standard TOPSIS method, the ratings and the weights of criteria are known precisely and it used crisp data to model real-world situations. However, in most situations, it is impractical to use crisp data model such situations. For example, human judgements or preferences are often vague and cannot estimate such preferences in exact numerical form [7]. The method SAW is one of the simplest, natural and most widely used multi-criteria evaluation methods. However, SAW uses only maximizing evaluation criteria, while minimizing evaluation criteria should be converted into the maximizing ones by the respective formulas prior to their application [8].

## 2. Preliminaries

### 2.1 Topologial indices

For a simple and finite graph *J* with vertex set *V*(*J*) and edge set *E*(*J*),some of the indices are defined as follows: The Zagreb Indices, *M*_1_(*J*) and *M*_2_(*J*), are topological indices that represent the sum of squared vertex degrees and the sum of squared vertex degrees of all edges in the molecular graph, respectively. Hyper Zagreb Index *HZ*(*J*) is defined as the sum of the squares of the degrees of the edges in a graph [9].

$$\begin{array}{c} M_{1}\left(J\right)=\sum_{uv\in E(J)}d\left(u\right)+d\left(v\right) \end{array}$$ (1)

$$\begin{array}{c} M_{2}\left(J\right)=\sum_{uv\in E(J)}d\left(u\right)\cdot d\left(v\right) \end{array}$$ (2)

$$\begin{array}{c} HZ(J)=\sum_{uv\in E(J)}{\left(d\left(u\right)+d\left(v\right)\right)}^{2} \end{array}$$ (3)

where *d*(*u*) and *d*(*v*) are the degrees of vertices *u* and *v*, respectively.

The Harmonic Index [10] of a graph is a measure of its structural complexity, particularly in the context of chemical graphs. It quantifies the deviation of a graph from being a tree-like structure, with higher values indicating greater complexity, the Harmonic Index *H*(*J*) is defined as:

$$\begin{array}{c} H\left(J\right)=\sum_{uv\in E(J)}\frac{2}{d(u)+d(v)} \end{array}$$ (4)

One of the most important indices while explaining property and structure relation, is Forgotten Index *F*(*J*). It is defined as the sum of the squares of the vertex degrees of a graph *J* [11].

$$\begin{array}{c} F\left(J\right)=\sum_{uv\in E(J)}\left(d{\left(u\right)}^{2}+d{\left(v\right)}^{2}\right) \end{array}$$ (5)

[12] introduced the Forgotten Index and explored few ground properties of the Forgotten Index. It also presented the role of the index in the Zagreb Index’s physical-chemical applicability.

The Sum Connectivity Index SCI(J) is a topological index used in graph theory to characterize the structural properties of a graph *J*.

It is defined as the sum of the numbers (*du* + *dv*)^−1/2^ over all edges uv of a graph *J* [12].

$$\begin{array}{c} SCI\left(J\right)=\sum_{uv\in E(J)}{\left(d\left(u\right)+d\left(v\right)\right)}^{-1/2} \end{array}$$ (6)

### 2.2 AD’s drugs structures

**Fig 1:**
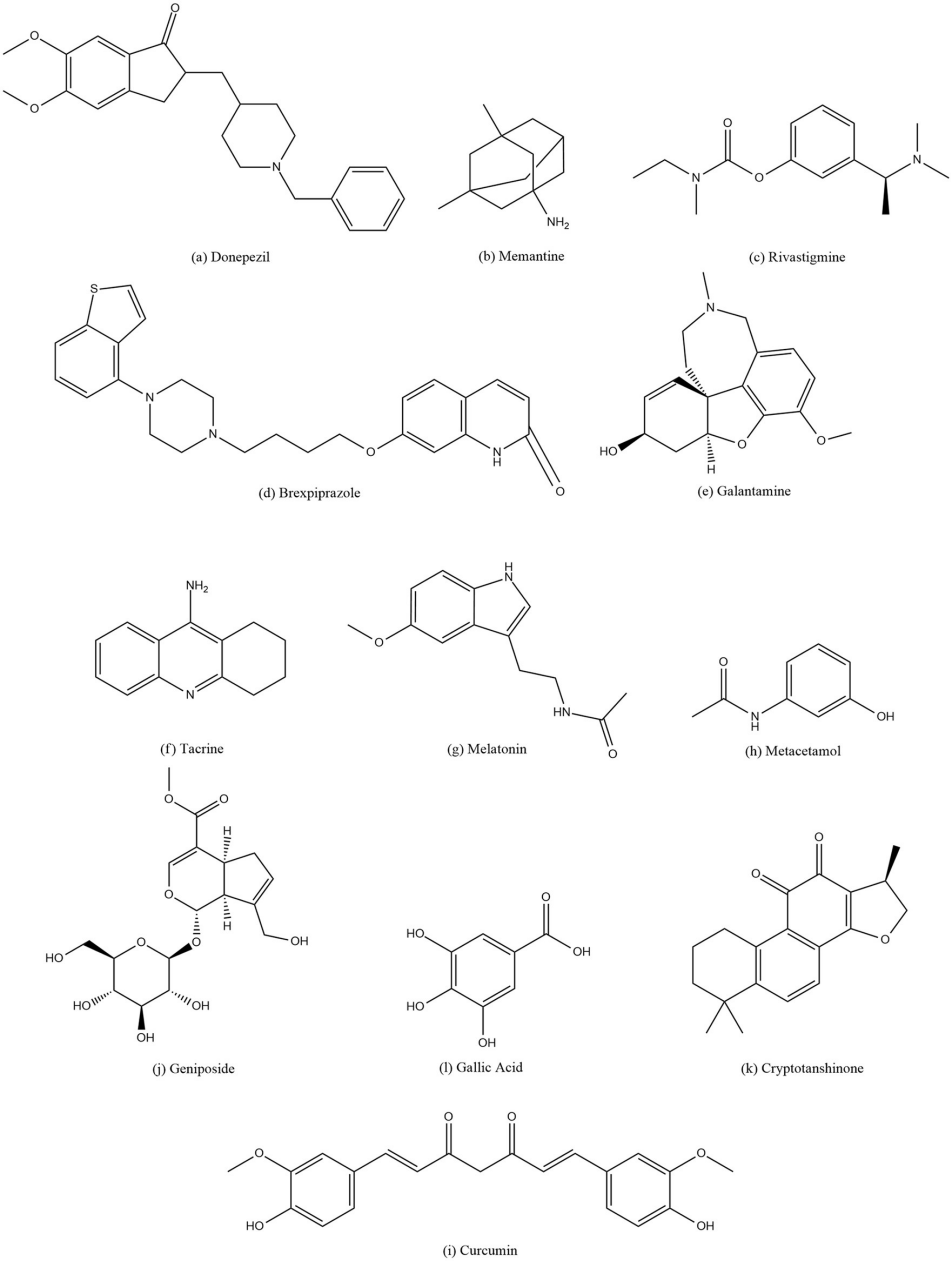


This research paper examines various molecular structures of AD’s drugs and their corresponding physiochemical characteristics, including melting point, boiling point, molecular weight, density, flash point and complexity. One of these drugs is Memantine, which is an N-Methyl-D-Aspartate (NMDA) receptor antagonist prescribed for AD and dementia [13]. Fig 1 depicts the structures of all the under consideration drugs.

Rivastigmine and Donepezil are cholinesterase inhibitor utilized for the treatment of AD and Parkinson’s disease dementia [14, 15]. Brexpiprazole is an atypical antipsychotic approved for the treatment of schizophrenia and major depressive disorder [16]. Galantamine is also a cholinesterase inhibitor and an allosteric modulator of nicotinic acetylcholine receptors, employed in AD’s therapy. Tacrine is used for AD, it imroves thinking ability [17]. Melatonin is a hormone that regulates sleep-wake cycles and is used as a supplement for sleep disorders and jet lag [18]. Metacetamol is a paracetamol (acetaminophen) prodrug used for pain relief and fever reduction [19]. Curcumin is a polyphenolic compound found in turmeric with antioxidant and anti-inflammatory properties, studied for various health benefits [20]. Geniposide is a natural compound with potential neuroprotective effects and anti-inflammatory properties [21].

Cryptotanshinone is a bioactive compound isolated from Salvia miltiorrhiza with anticancer and anti-inflammatory properties [22]. Gallic acid is a natural antioxidant found in various plants, fruits, and herbs, known for its anti-inflammatory and anti-tumor effects [23].

In this research project, a comprehensive evaluation of various topological indices will be conducted. The aim is to utilize topological invariants to provide a more cost-effective and efficient method for pharmacists to determine the physio-chemical characteristics of AD’s drugs. Two different MCDM techniques, namely TOPSIS and SAW, will be employed to carry out this evaluation. The Entropy method will be utilized for assigning appropriate weights to the criteria based on the physical properties.

## 3. Methodology

### 3.1 Calculation of topological indices

We have calculated the topological indices of an AD’s drug structure *Donepezil* having the edge partition,

|*E*_1,2_| = 2, |*E*_1,3_| = 1, |*E*_2,2_| = 6, |*E*_2,3_| = 18, |*E*_3,3_| = 4
*M*_1_(*Donepezil*) = 2(3) + 1(4) + 6(4) + 18(5) + 4(6) = 148
*M*_2_(*Donepezil*) = 2(2) + 1(3) + 6(4) + 18(6) + 4(9) = 175

$H\left(Donepezil\right)=2\left(\frac{2}{3}\right)+21\frac{2}{4})+6\left(\frac{2}{4}\right)+18\left(\frac{2}{5}\right)+4\left(\frac{2}{6}\right)=13.36667$

*F*(*Donepezil*) = 2(5) + 1(10) + 6(8) + 18(13) + 4(18) = 374
*HZ*(*Donepezil*) = 2(3)^2^ + 1(4)^2^ + 6(4)^2^ + 18(5)^2^ + 4(6)^2^ = 724
*SCI*(*Donepezil*) = 2(3)^−1/2^ + 1(4)^−1/2^ + 6(4)^−1/2^ + 18(5)^−1/2^ + 4(6)^−1/2^ = 14.3375

**Fig 2:**
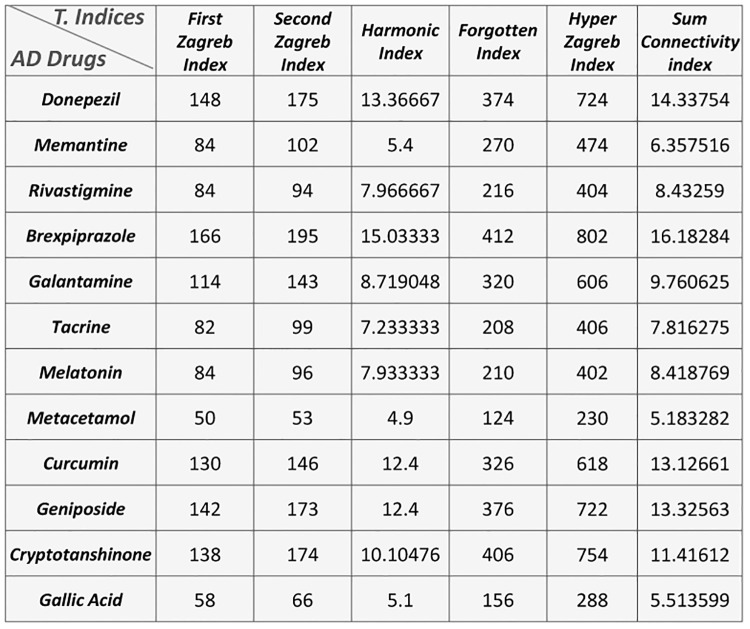
AD’s topological indices.

All the remaining topological indices of each drug are calculated in similar way and numerical results of topological indices are mentioned in Fig 2.

Now before applying TOPSIS and SAW, two more things are still there to reflect light upon them. One of them is weight allocation of indices as explained in next section and the second one is the decision for ideal best and ideal worst cases for drugs.

### 3.2 Entropy method for weight allocation

For the application of TOPSIS and SAW [24], weight allocation to the topological alternatives is required. For required weight calculation, the Entropy method is employed. The Entropy method is an MCDM technique that helps decision-makers prioritize alternatives based on their Entropy values, which reflect the uncertainty or variability associated with the criteria [25, 26]. After obtaining topological indices, the Entropy method is employed in calculating weights for drug alternatives. The Entropy method is widely employed as an objective approach to assign weights to criteria or constructs [27]. It calculates objective weights for attributes or responses, quantifying their importance without incorporating the preferences of decision-makers. By measuring the uncertainty of variables, Entropy assesses how influencing factors affect outcomes. This method provides a quantitative measure of information content, facilitating comparisons and analyses across various statistical models, algorithms, and their tuning parameters [28].

#### 3.2.1 Establish the decision matrix (*D*_*ij*_)

**Fig 3:**
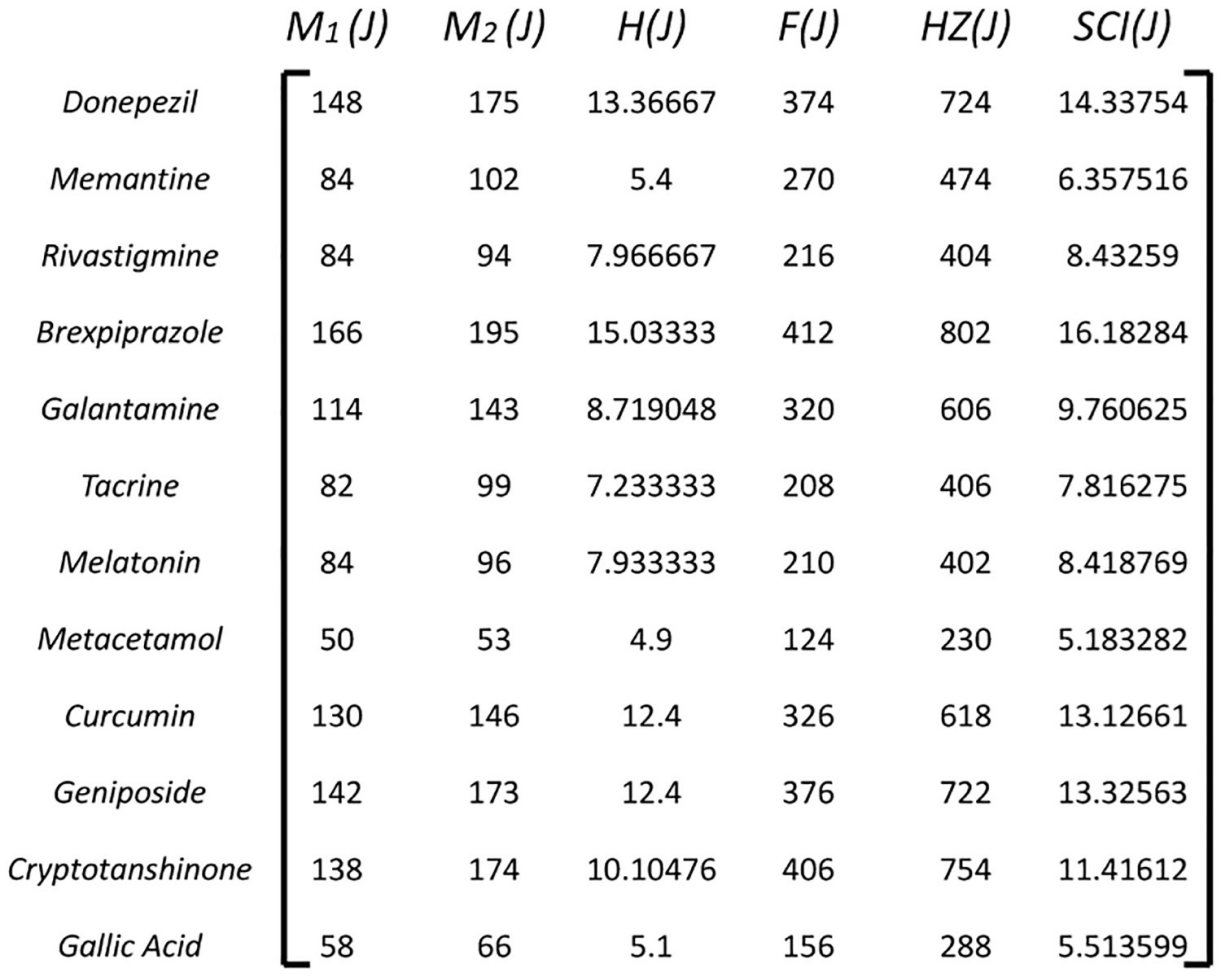
Decision matrix *D_ij_*.

Construct a decision matrix where rows represent the 12 drug alternatives and columns represent the criteria of topological indices. Populate the matrix with data reflecting the performance of each drug alternative on each topological index as in Fig 3.

#### 3.2.2 Calculate Normalized Decision Matrix(*ND*_*ij*_)

**Fig 4:**
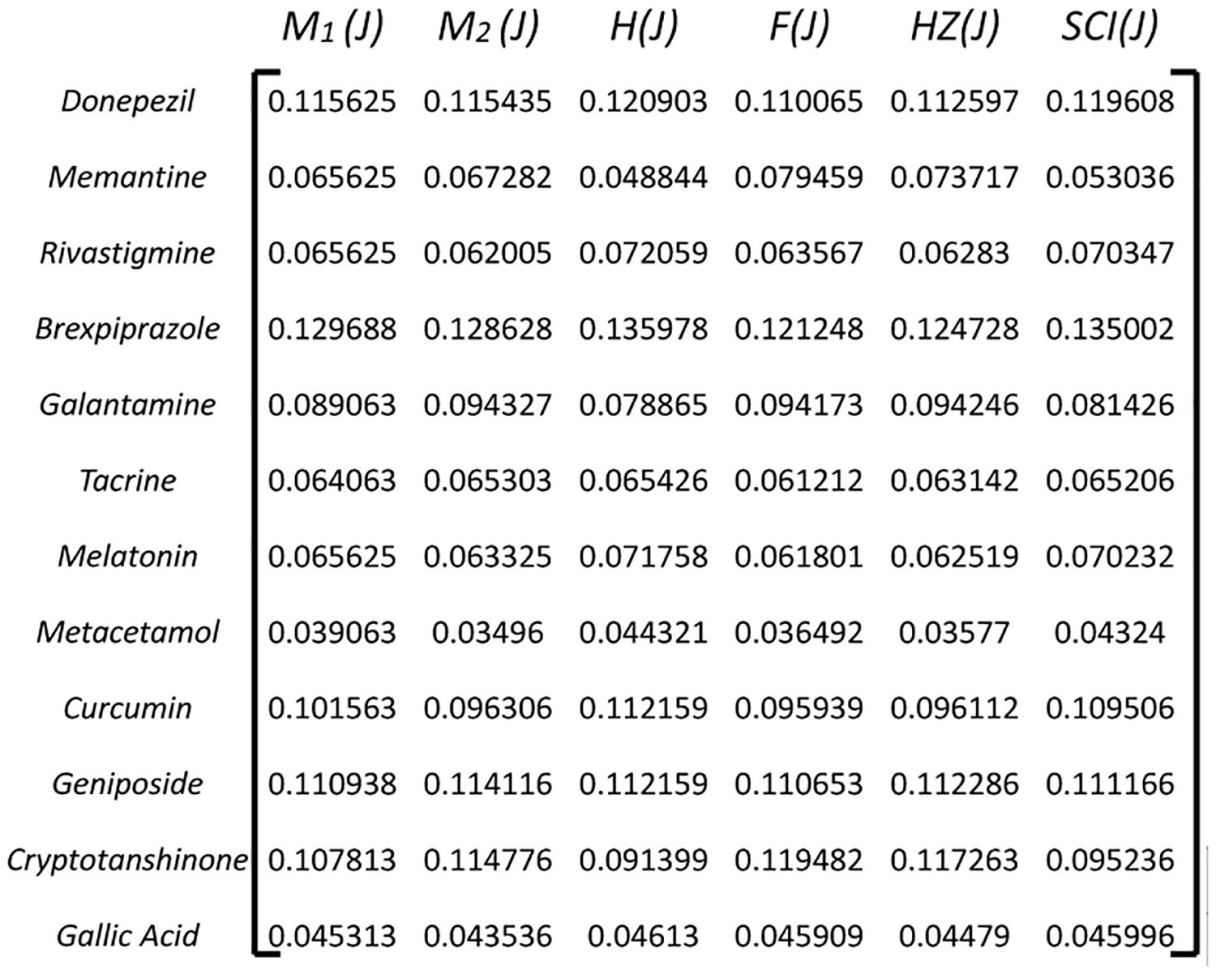
Normalized decision matrix *H*_*ij*_.

Normalize the decision matrix to bring all criteria onto a common scale. This is typically done by dividing each element in the matrix by the sum of the column it belongs to. Normalization ensures that criteria with different units or scales are treated equally as shown in Fig 4.

$$\begin{array}{c} \left[r_{ij}\right]=\frac{x_{ij}}{\sum_{j=1}^{n}x_{ij}} \end{array}$$ (7)

where

$$\begin{array}{c} i=1,2,...m,j=1,...n \end{array}$$

#### 3.2.3 Compute entropy for each criterion

Calculate the Entropy for each criterion to measure its uncertainty or variability. Entropy is calculated using the formula:

$$\begin{array}{c} \left[k_{ij}\right]=r_{ij}\;\text{ln}\left(r_{ij}\right) \end{array}$$ (8)

$$\begin{array}{c} E_{ij}=-h\sum_{i=1}^{m}r_{ij}\;\text{ln}\left(r_{ij}\right) \end{array}$$ (9)

Where $h=\frac{1}{ln(m)}$, *E*_*ij*_ is the Entropy of criterion *i* and *r*_*ij*_ is the normalized value of alternative *j* on criterion *i*.

#### 3.2.4 Determine weight of each criterion

Weight each criterion based on its Entropy value. Higher Entropy values indicate greater uncertainty, and thus, less informative criteria should be assigned lower weights. The weight of each criterion is calculated as:

$$\begin{array}{c} W_{j}=\frac{1-E_{ij}}{\sum_{j=1}^{n}\left(1-E_{ij}\right)} \end{array}$$ (10)

**Fig 5:**
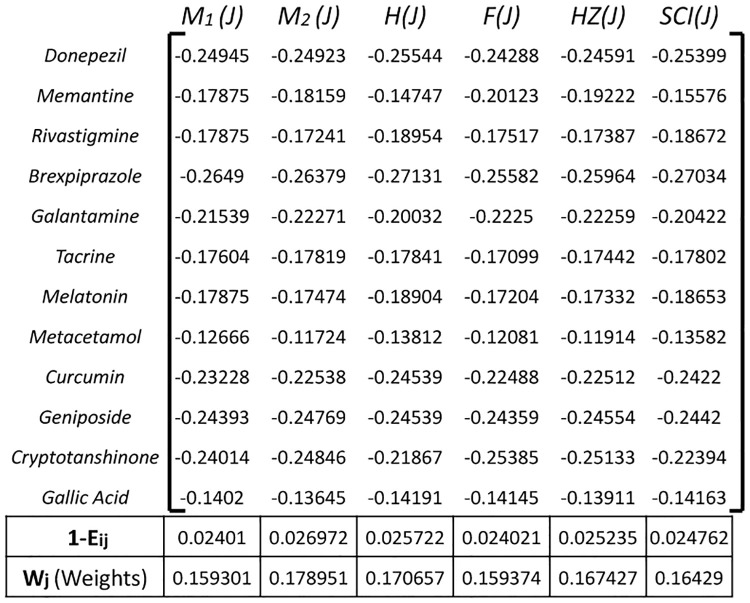
Entropy and weight calculations.

Fig 5 indicates the Entropy for each criteria along with final weight calculations. These weights are used in the application of both TOPSIS and SAW methods.

### 3.3 Ideal best and ideal worst for physical properties

Before moving on, we need to decide on a drug’s physiochemical property, and whether it has a positive or negative effect. The question is “What is the Ideal Best or Ideal Worst case for a physical property of a certain drug?”. Some common and impactful properties are considered in this study, i.e., boiling point, melting point, molecular weight, density, flash point, and complexity. Physical properties and topological indices are correlated using Statistical Package for Social Science (SPSS) [29]. Based on these correlation results, physical properties are allocated to indices. For further Ideal Best and Ideal Worst discussion, each criterion is evaluated. For the boiling point of a drug structure, the higher the boiling point is, the less volatile it is. Drugs with higher boiling points are more considerable [30]. Melting point is also a basic property of any drug in medical sciences. Lower melting point indicates higher absorption [31]. Another major property is the molecular weight. Higher molecular weight results in a higher absorption degree. Lower density of a drug is preferred because higher density substances will be resistant to absorption [32]. Another considerable attribute is the flash point. Materials with higher flash points are less flammable or hazardous than chemicals with lower flash points [31]. Lastly, the complexity of a drug is acknowledged to be a risk factor for administration errors and nonadherence, resulting in increased healthcare expenses [33].

### 3.4 Ranking ADs drugs using TOPSIS

This method of MCDM first appeared in the 1980s. TOPSIS will be the first technique used in our study for evaluation. This weighted evaluation will be carried out for the ideal solution and the greatest distance from the worst solution [24]. It also tries to use mathematics to assess the accuracy of molecular compound specifications.

#### 3.4.1 Normalization

**Fig 6:**
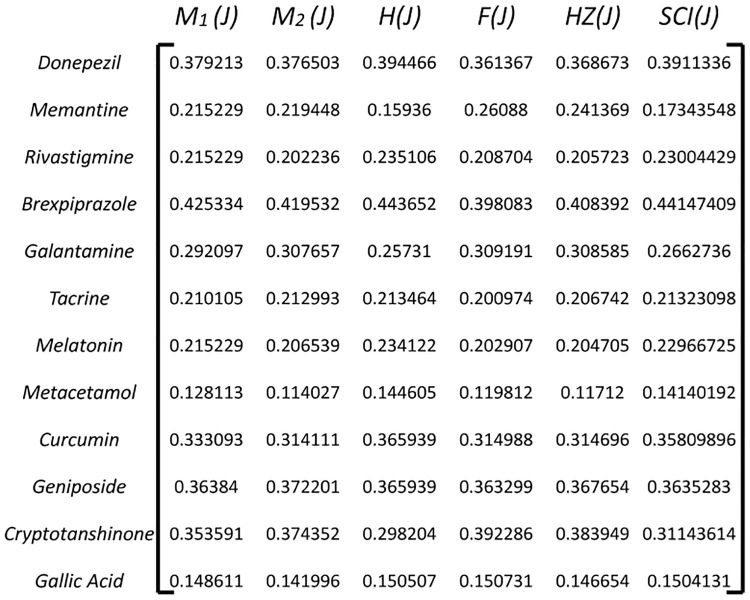
Normalized decision matrix *H*_*ij*_.

The first step is to normalize the decision matrix, which contains the performance of each alternative across all criteria. This step ensures that all criteria are on the same scale and allows for fair comparison as shown in Fig 6.

$$\begin{array}{c} \text{Normalized}\;\text{Value}\left(r_{ij}\right)=\frac{x_{ij}}{\sqrt{\sum_{i=1}^{m}x_{ij}^{2}}} \end{array}$$

#### 3.4.2 Weighting

**Fig 7:**
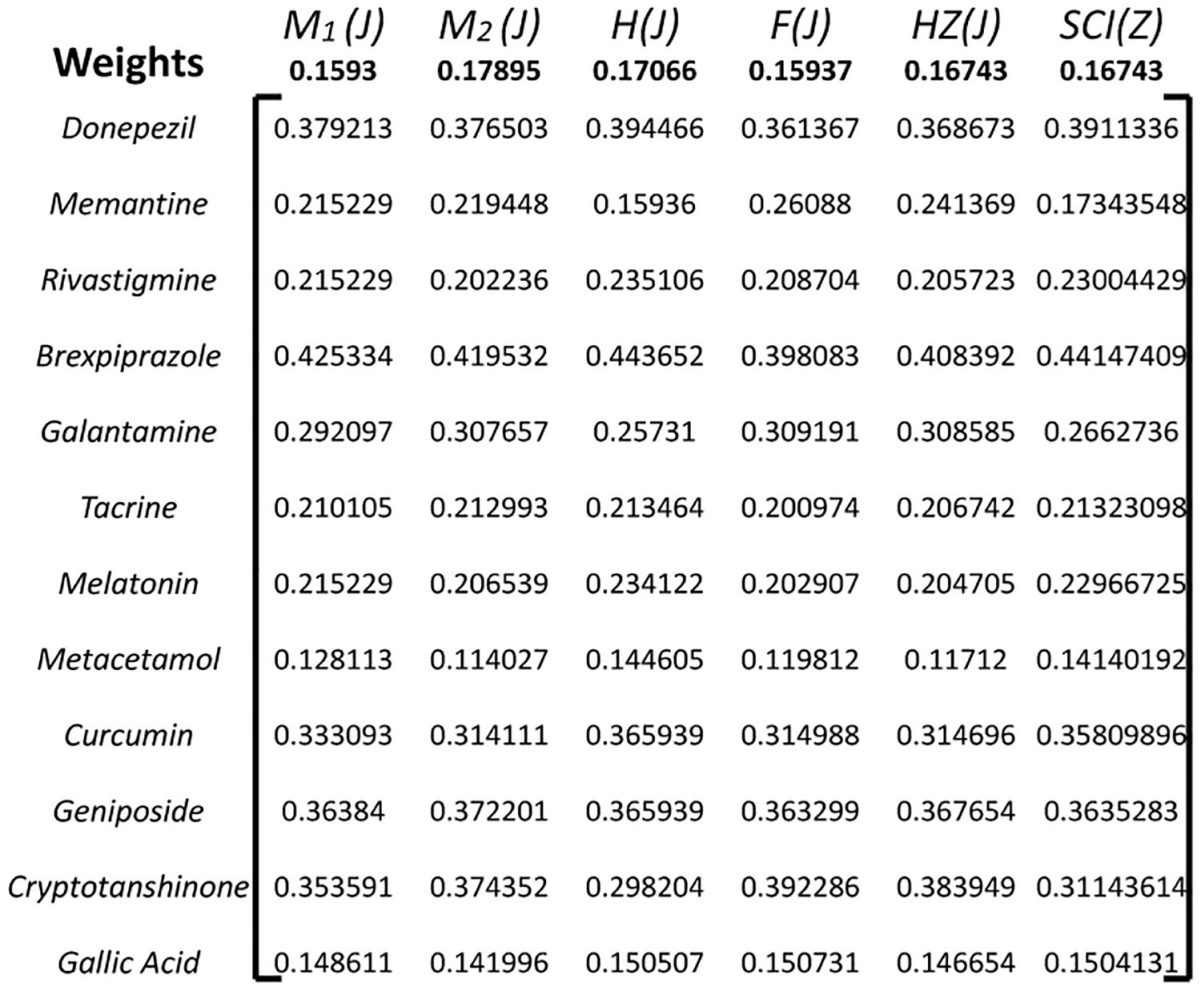
Weighted normalized decision matrix *X*_*ij*_.

Next, weights are assigned to each criterion to reflect their relative importance in the decision-making process. These weights can be determined based on the preferences of decision-makers or through other methods such as the Entropy method as in Fig 7.

$$\begin{array}{c} \text{Weighted}\;\text{Normalized}\;\text{Value}\left(v_{ij}\right)=w_{j}\times r_{ij} \end{array}$$

#### 3.4.3 Ideal and anti-ideal solutions

**Fig 8:**
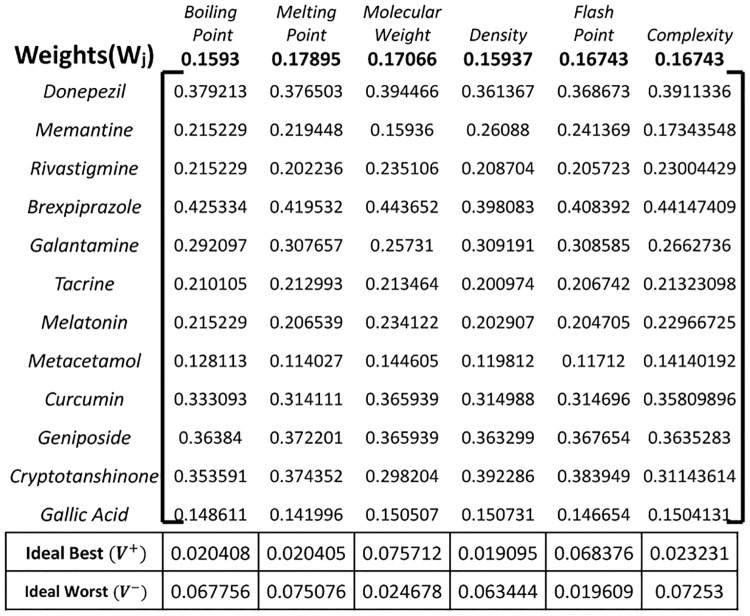
Calculation of separating measures $P_{i}^{+}$ and $P_{i}^{-}$.

The ideal solution represents the best performance across all criteria, while the anti-ideal solution represents the worst performance. These solutions are calculated based on the maximum and minimum values for each criterion, respectively in Fig 8.

$$\begin{array}{c} \text{Ideal}\;{\text{Solution}}^{+}=(\overset{n}{\underset{j=1}{\text{max}}}\;v_{ij}) \end{array}$$

$$\begin{array}{c} \text{Anti-Ideal}\;{\text{Solution}}^{-}=(\overset{n}{\underset{j=1}{\text{min}}}\;v_{ij}) \end{array}$$

#### 3.4.4 Similarity calculation

The similarity of each alternative to the Ideal and Anti-Ideal solutions is then calculated using a distance metric, typically the Euclidean distance or another distance measure such as the Manhattan distance.

$$\begin{array}{c} P^{+}\left(A_{i}\right)=\sqrt{\sum_{j=1}^{n}{\left(v_{ij}-\text{Ideal}\;{\text{Solution}}_{j}^{+}\right)}^{2}}, \end{array}$$ (11)

$$\begin{array}{cc} P^{-}\left(A_{i}\right) & =\sqrt{\sum_{j=1}^{n}{\left(v_{ij}-\text{Anti-Ideal}\;{\text{Solution}}_{j}^{-}\right)}^{2}}. \end{array}$$ (12)

#### 3.4.5 Relative closeness

**Fig 9:**
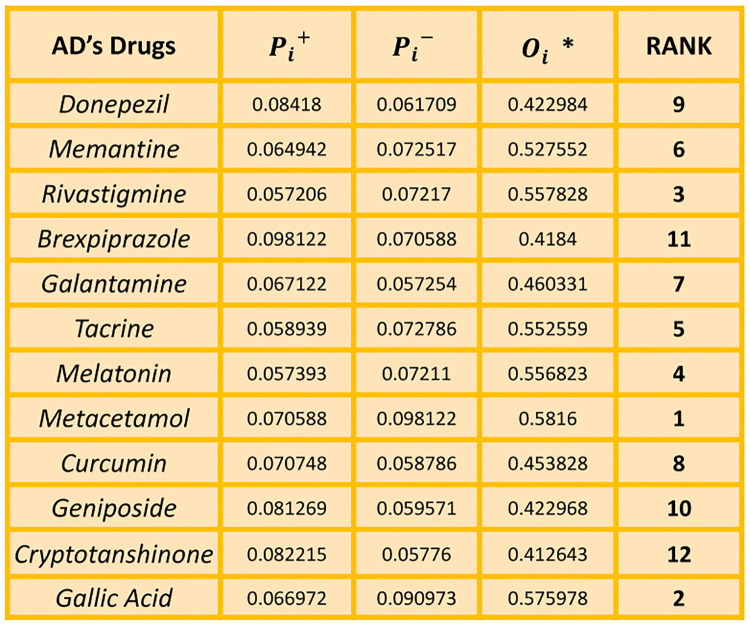
Calculation of Ideal measures $O_{i}^{*}$ and ranking of drugs using TOPSIS.

Finally, the relative closeness of each alternative to the Ideal solution is determined by comparing its distance to the Ideal solution with its distance to the Anti-Ideal solution. Alternatives with shorter distances to the Ideal solution and longer distances to the Anti-Ideal solution are considered more preferable and are ranked higher in Fig 9.

$$\begin{array}{cc} Closeness(O_{i}^{*}) & =\frac{P^{-}\left(A_{i}\right)}{P^{+}\left(A_{i}\right)+P^{-}\left(A_{i}\right)} \end{array}$$ (13)

### 3.5 Ranking ADs drugs using SAW

SAW is a widely used MCDM method that involves assigning weights to criteria and then summing up the weighted scores of alternatives to rank them [24]. Here’s how SAW works:

#### 3.5.1 Normalization

The first step is to normalize the decision matrix, which contains the performance of each alternative across all criteria. Normalization ensures that all criteria are on the same scale and allows for fair comparison.

#### 3.5.2 Weighting

Next, weights are assigned to each criterion to reflect their relative importance in the decision-making process. These weights can be determined based on the preferences of decision-makers, stakeholder consultations, or other methods such as the Entropy method.

$$\begin{array}{c} \text{Weighted}\;\text{Score}\left(S_{ij}\right)=x_{ij}\times w_{j} \end{array}$$

#### 3.5.3 Scoring

Each alternative is scored on each criterion by multiplying its performance value by the corresponding weight. This results in a weighted score for each alternative on each criterion.

$$\begin{array}{c} \text{Total}\;\text{Score}\left(TS_{i}\right)=\sum_{j=1}^{n}S_{ij} \end{array}$$

#### 3.5.4 Aggregation

The weighted scores for each alternative are then aggregated by summing them up across all criteria. This results in a total score for each alternative.

#### 3.5.5 Ranking

Finally, the alternatives are ranked based on their total scores, with higher scores indicating better performance.

**Fig 10:**
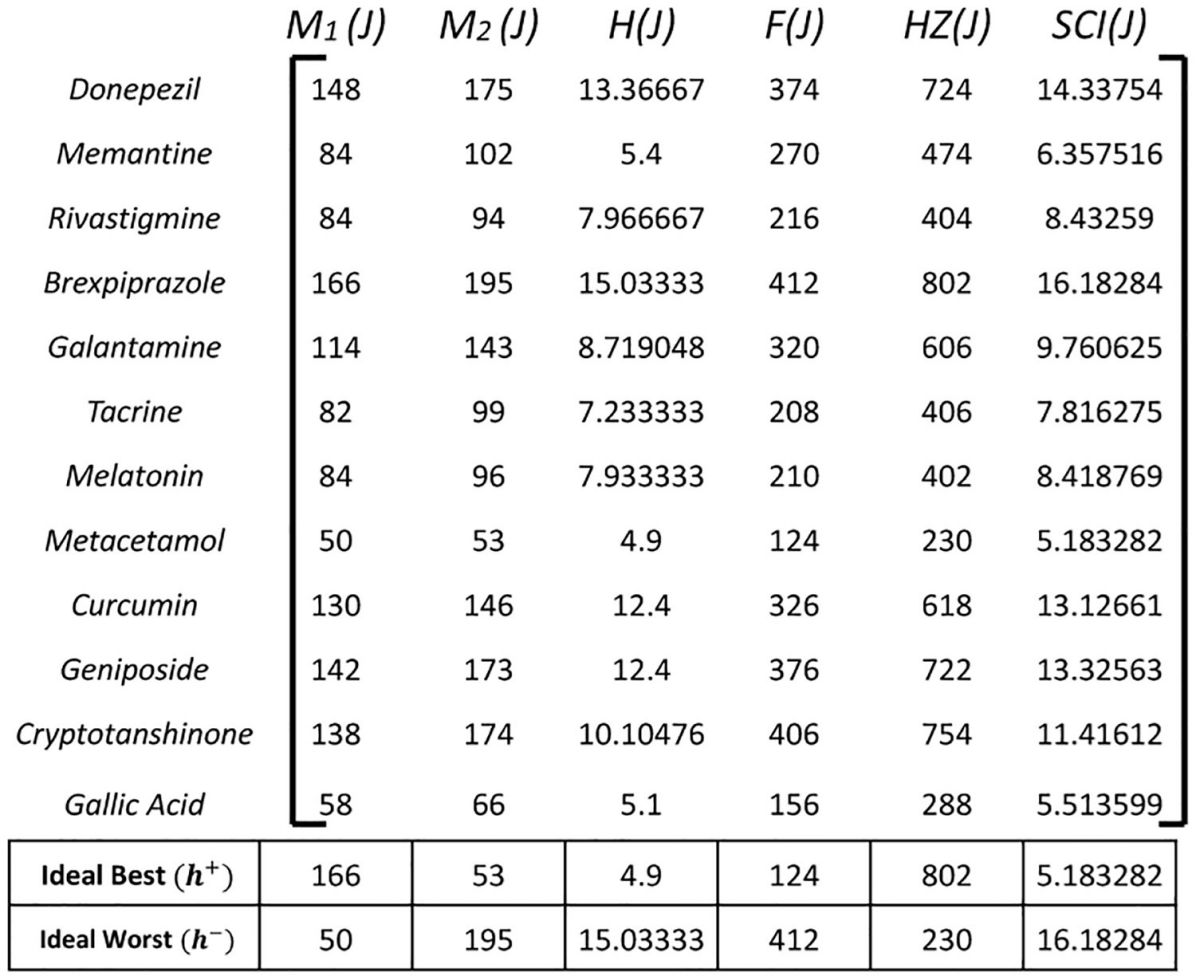
Decision matrix *D*_*ij*_ for SAW.

**Fig 11:**
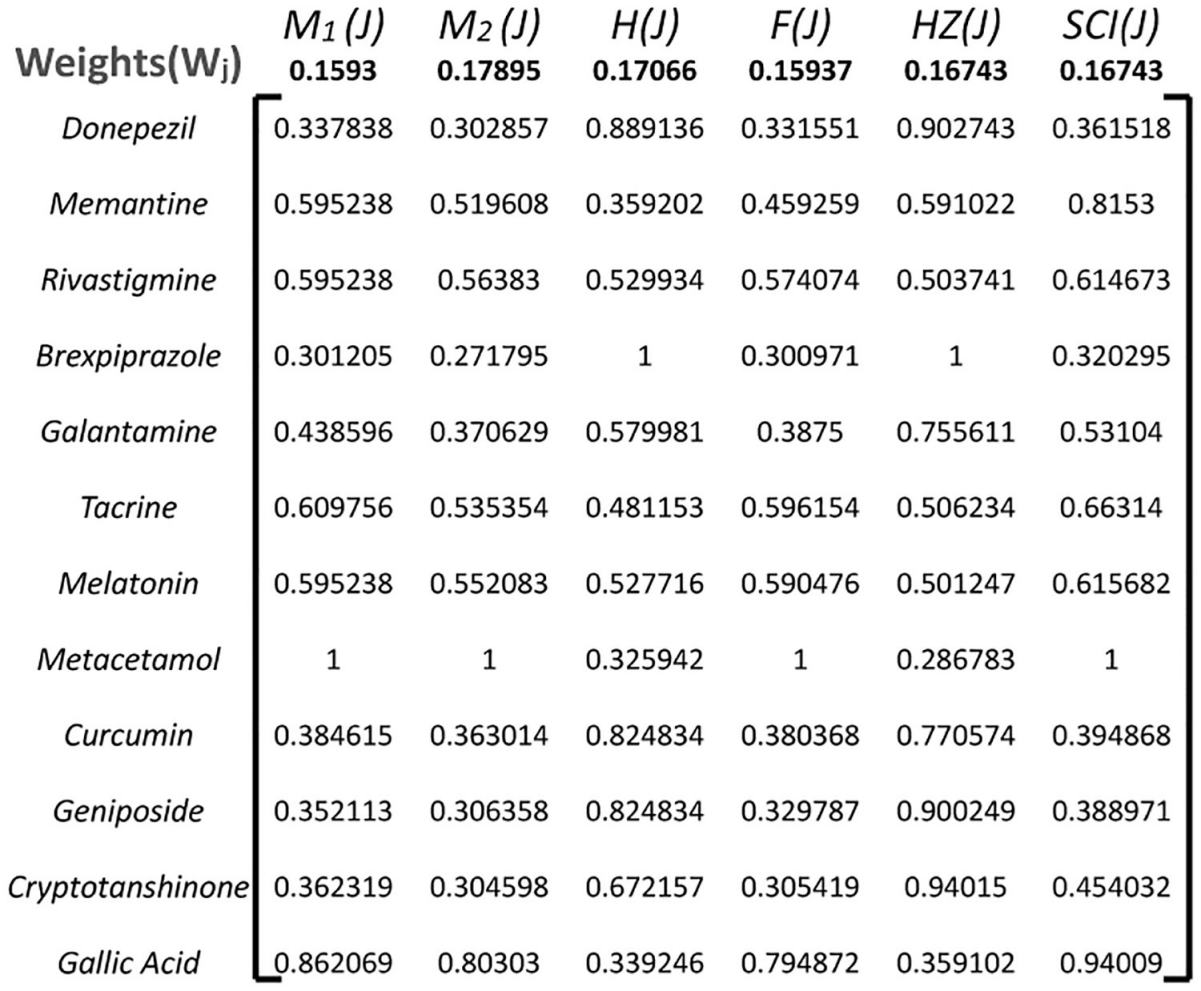
Weighted normalized decision matrix *X*_*ij*_ for SAW.

**Fig 12:**
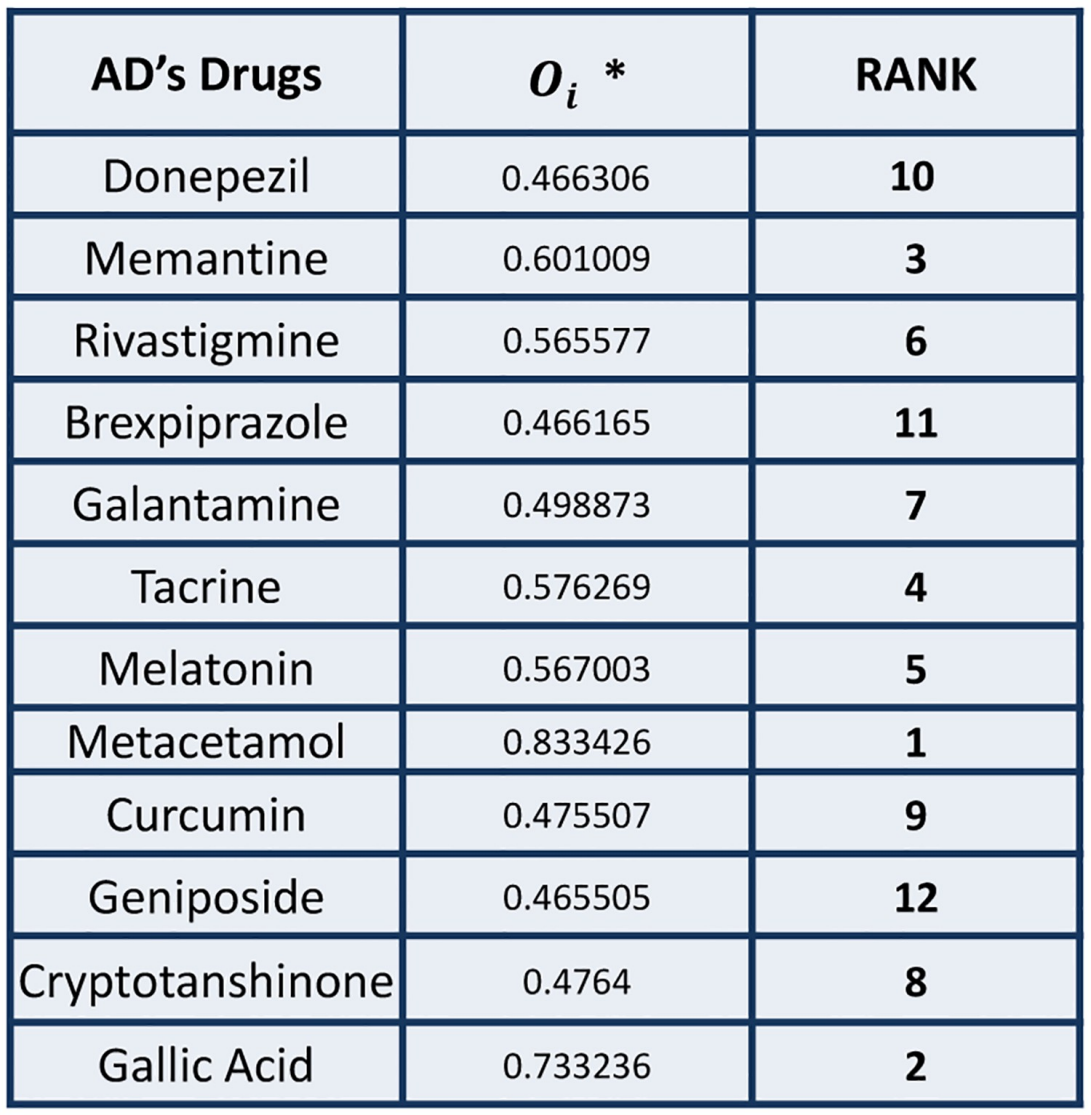
Calculation of ideal measures $O_{i}^{*}$ and Ranking of Drugs using SAW.

SAW is straightforward and easy to implement, making it a popular choice for decision-making in various fields such as business, finance, and project management. However, it does not account for interactions between criteria and may not always produce the most optimal solution. Figs 10–12 represents the outcomes of SAW method.

## 4. Conclusion

**Fig 13:**
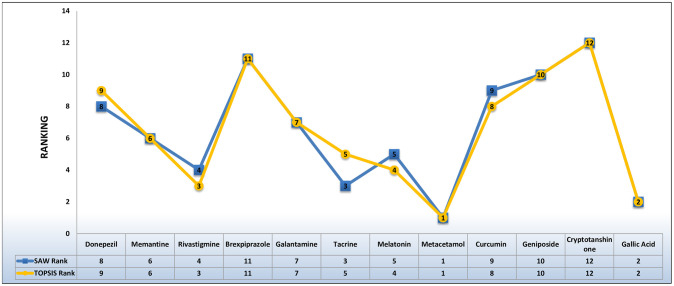


In this study, 12 AD drugs have been ranked based on their QSPR analysis. This analysis has been done by employing two MCDM techniques namely TOPSIS and SAW while using the Entropy method for weight allocation. Fig 13 represents the predicted ranking of the drugs. Metacetamol is proved to be the best drug as it is the closest to the ideal solution in both cases. Galli acid, Memantine, Galantamine, Geniposide, and Cryptotanshinone have the same ranks in both respective methods. These rankings may be fruitful for researchers in the field of pharmaceutical and medicinal sciences.

There is an important limitation to this type of study. The results cannot be compared with a ‘gold standard’ of what the best AD drug actually is or what the order of preference for the different drug classes should be. However, the pharmaceutical sector will be able to produce fresh treatments that will undoubtedly be beneficial in acquiring preventive measures for the aforementioned sickness with the calculated value derived from this. They provide techniques for estimating attributes for fresh exposures to various diseases. It will be useful in determining and forecasting a variety of properties and processes, such as Entropy, critical pressure, boiling point, acentric factor, enthalpy, and others. Our discoveries may also help in the development of new medications for the treatment of Alzheimer.

## References

[1] Alzheimer’s Association. (2022). Alzheimer’s disease facts and figures. Alzheimer’s and Dementia, 18(1), e1–e98. 10.1002/alz.12638 35289055

[2] Cummings J., Lee G., Ritter A., and Zhong K. (2021). Alzheimer’s disease drug development pipeline: Alzheimer’s and Dementia: Translational Research and Clinical Interventions, 7(1), e12179. 10.1002/trc2.12179 PMC8145448 34095440

[3] Selkoe D. J., and Hardy J. (2016). The amyloid hypothesis of Alzheimer’s disease at 25 years. EMBO Molecular Medicine, 8(6), 595–608. doi: 10.15252/emmm.201606210 27025652 PMC4888851

[4] Todeschini, R., Consonni, V. (2000). Handbook of molecular descriptors. Wiley-VCH.

[5] Roy K., Mitra I., and Kar S. (2015). Topological descriptors in drug discovery and development. Progress in Biophysics and Molecular Biology, 118(1-2), 22–43.

[6] Bondy, J. A., and Murty, U. S. R. (2008). Graph theory. Springer Science and Business Media, 244.

[7] Madi, E. N., Garibaldi, J. M., and Wagner, C. (2016). An exploration of issues and limitations in current methods of TOPSIS and fuzzy TOPSIS. IEEE International Conference on Fuzzy Systems, 2098–2105.

[8] Podvezko V. (2011). The comparative analysis of MCDA methods SAW and COPRAS. Engineering Economics, 22(2), 134–146. doi: 10.5755/j01.ee.22.2.310

[9] Diestel, R. (2005). Graph theory. Springer, 173.

[10] Gutman I. (1978). The energy of a graph. Berichte der Mathematisch-Statistischen Sektion im Forschungszentrum Graz, 1(2), 1–22.

[11] Randić M. (1975). Characterization of molecular branching. Journal of the American Chemical Society, 97(23), 6609–6615. doi: 10.1021/ja00856a001

[12] De N., Nayeem S. M. A., and Pal A. (2016) F-index of some graph operations. Discrete mathematics, algorithms and applications, 8(2), 1650025. doi: 10.1186/s40064-016-1864-7 27026915 PMC4771675

[13] Reisberg B., Doody R., Stöffler A., Schmitt F., Ferris S., and Möbius H. J. (2003). Memantine in moderate-to-severe Alzheimer’s disease. New England Journal of Medicine, 348(14), 1333–1341. doi: 10.1056/NEJMoa013128 12672860

[14] Salloway S., Sperling R., Fox N. C., Blennow K., Klunk W., Raskind M., et al. (2014). Two phase 3 trials of bapineuzumab in mild-to-moderate Alzheimer’s disease. New England Journal of Medicine, 370(4), 322–333. doi: 10.1056/NEJMoa1304839 24450891 PMC4159618

[15] Emre M., Aarsland D., Albanese A., Byrne E. J., Deuschl G., De Deyn P. P., et al. (2004). Rivastigmine for dementia associated with Parkinson’s disease. New England Journal of Medicine, 351(24), 2509–2518. doi: 10.1056/NEJMoa041470 15590953

[16] Citrome L., Earley W., and Szatmári B. (2016). Brexpiprazole for the treatment of schizophrenia: A review of this novel serotonin–dopamine activity modulator. Clinical Schizophrenia and Related Psychoses, 10(4), 190–199. 26757416

[17] Wilcock G. K., Lilienfeld S., Gaens E., and Nicholson J. P. (2000). Tacrine: a 10-year overview. Alzheimer Disease and Associated Disorders, 14(4), 188–198.

[18] Auld F., Maschauer E. L., Morrison I., Skene D. J., and Riha R. L. (2010). Evidence for the efficacy of melatonin in the treatment of primary adult sleep disorders. Sleep Medicine Reviews, 14(6), 397–405. 28648359 10.1016/j.smrv.2016.06.005

[19] Vaysse P. M., Gardner I., Tucker G. T., Rostami-Hodjegan A., Nicholson J. K., Andreu F., et al. (2018). Physiologically based pharmacokinetic modeling of a novel paracetamol prodrug: A tale on the importance of in vitro-in vivo extrapolation and simulation strategy. Drug Metabolism and Disposition, 46(6), 764–776.

[20] Hewlings S. J., and Kalman D. S. (2017). Curcumin: A review of its effects on human health. Foods, 6(10), 92. doi: 10.3390/foods6100092 29065496 PMC5664031

[21] Fu R. H., Hranjec T., Meng T. J., Wang Y. K., Guo L. Y., Remick D. G., et al. (2018). Cryptotanshinone protects against systemic inflammation-induced memory impairment via TLR4/Myd88/NF-KB signaling pathway in mice. International Immunopharmacology, 59, 24–30.

[22] Lee, K. Y., Kim, S. I., Jung, S. H., Kim, J. H., Kim, Y. W., Hwang, S. Y., et al. (2018). Anti-inflammatory and neuroprotective effects of constituents isolated from Rhodiola rosea. Evidence-Based Complementary and Alternative Medicine. 10.1155/2013/514049 PMC3652169 23690847

[23] Salehi B., Fokou P. V. T., Yamthe L. R. T., Tali B. T., Adetunji C. O., Rahavian A., et al. (2019). Phytochemicals in Helicobacter pylori infections: What are we doing now? International Journal of Molecular Sciences, 20(24), 6056. 30103451 10.3390/ijms19082361 PMC6121492

[24] Ciardiello F., and Genovese A. (2023). A comparison between TOPSIS and SAW methods. Annals of Operations Research, 325(2), 967–994. doi: 10.1007/s10479-023-05339-w

[25] Keeney, R. L., and Raiffa, H. (1993). Decisions with multiple objectives: Preferences and value trade-offs. Cambridge University Press.

[26] Saaty, T. L. (1980). The Analytic Hierarchy Process: Planning, Priority Setting, Resource Allocation. McGraw-Hill.

[27] Teixeira S. J., Ferreira J. J., Wanke P., and Moreira Antunes J. J. (2021). Evaluation model of competitive and innovative tourism practices based on information entropy and alternative criteria weight. Tourism Economics, 27(1), 23–44. doi: 10.1177/1354816619878995

[28] Mukhametzyanov I. (2021). Specific character of objective methods for determining weights of criteria in MCDM problems: Entropy, CRITIC and SD. Decision Making: Applications in Management and Engineering, 4(2), 76–105.

[29] Kafle S. C. (2019). Correlation and regression analysis using SPSS. Management, Technology and Social Sciences, 126.

[30] Kanwal, S., Farooq, Y., Siddiqui, M. K., Idrees, N., Razzaque, A., and Petros, F. B. (2023). Study the Behavior of Drug Structures via Chemical Invariants Using TOPSIS and SAW. Computational and Mathematical Methods in Medicine. 10.1155/2023/4262299 PMC9902132 36756388

[31] Bokhary S. A. U. H., Adnan, Siddiqui M. K., and Cancan M. (2022). On topological indices and QSPR analysis of drugs used for the treatment of breast cancer. Polycyclic Aromatic Compounds, 42(9), 6233–6253.

[32] Abramowitz R., and Yalkowsky S. H. (1990). Melting point, boiling point, and symmetry. Pharmaceutical research, 7, 942–947. doi: 10.1023/A:1015949907825 2235894

[33] Sen L., Yang Z., Caihong Z., and Chengliang W. (2021). A comprehensive evaluation of county economies in the Beijing-Tianjin-Hebei Region based on entropy TOPSIS analysis. Applied Mathematics and Nonlinear Sciences, 6(2), 499–516. doi: 10.2478/amns.2021.2.00014

